# Polymorphisms in DNA mismatch repair pathway genes predict toxicity and response to cisplatin chemoradiation in head and neck squamous cell carcinoma patients

**DOI:** 10.18632/oncotarget.25268

**Published:** 2018-07-03

**Authors:** Guilherme Augusto Silva Nogueira, Ericka Francislaine Dias Costa, Leisa Lopes-Aguiar, Tathiane Regine Penna Lima, Marília Berlofa Visacri, Eder Carvalho Pincinato, Gustavo Jacob Lourenço, Luciane Calonga, Fernanda Viviane Mariano, Albina Messias de Almeida Milani Altemani, João Maurício Carrasco Altemani, Patrícia Moriel, Carlos Takahiro Chone, Celso Dario Ramos, Carmen Silvia Passos Lima

**Affiliations:** ^1^ Department of Internal Medicine, Faculty of Medical Sciences, University of Campinas, Campinas, São Paulo, Brazil; ^2^ Department of Clinical Pathology, Faculty of Medical Sciences, University of Campinas, Campinas, São Paulo, Brazil; ^3^ Laboratory of Cancer Genetics, Faculty of Medical Sciences, University of Campinas, Campinas, São Paulo, Brazil; ^4^ Department of Ophthalmology and Otorhinolaryngology, Faculty of Medical Sciences, University of Campinas, Campinas, São Paulo, Brazil; ^5^ Department of Pathology, Faculty of Medical Sciences, University of Campinas, Campinas, São Paulo, Brazil; ^6^ Department of Radiology, Faculty of Medical Sciences, University of Campinas, Campinas, São Paulo, Brazil

**Keywords:** head and neck squamous cell carcinoma, mismatch repair pathway, single nucleotide polymorphism, outcome

## Abstract

Head and neck squamous cell carcinoma (HNSCC) is treated with cisplatin (CDDP) and radiotherapy (RT), and distinct results are observed among patients with similar clinicopathological aspects. This prospective study aimed to investigate whether *MLH1* c.-93G>A (rs1800734), *MSH2* c.211+9C>G (rs2303426), *MSH3* c.3133G>A (rs26279), *EXO1* c.1765G>A (rs1047840), and *EXO1* c.2270C>T (rs9350) single nucleotide polymorphisms (SNPs) of the mismatch repair (MMR) pathway change side effects and response rate of 90 HNSCC patients treated with CDDP and RT. DNA from peripheral blood was analyzed by PCR-based methods to obtain genotypes. It was observed 4.27-fold and 4.69-fold increased risks of presenting pronounced nephrotoxicity with treatment in patients with *MSH3* GG and *EXO1* rs9350 CC genotypes compared with patients with GA or AA and CT or TT genotypes, respectively. *MSH3* GG or GA and GT haplotype of *EXO1* rs1047840 and rs9350 SNPs conferred to patients 10.29 and 4.00 more chances of presenting pronounced ototoxicity after treatment than *MSH3* AA genotype and other *EXO1* haplotypes, respectively. Patients with *EXO1* rs1047840 GA or AA genotype and AC haplotype of *EXO1* rs1047840 and rs9350 SNPs had both 9.55-fold increased risks of achieving partial response or stable disease instead of complete remission after treatment than patients with *EXO1* GG genotype and other *EXO1* haplotypes, respectively. For the first time, our data show preliminary indication that inherited alterations of DNA MMR pathway, related to *MSH3* rs26279, *EXO1* rs1047840 and *EXO1* rs9350 SNPs, modify toxicity and response to chemoradiation in HNSCC, and may contribute to future personalized treatment of patients.

## INTRODUCTION

The treatment of advanced unresectable head and neck squamous cell carcinoma (HNSCC) has been made with association between cisplatin (CDDP) and radiotherapy (RT) [1]. CDDP binds to DNA, forming adducts, and it also favors accumulation of intracellular free radicals [2]. RT induces lesion to DNA, direct and indirectly, by activity of photons and free radical generation, respectively [3]. DNA damages induced by CDDP and RT trigger apoptosis when not properly repaired by DNA repair pathways, such as the mismatch repair (MMR) [4, 5]. MutL homolog 1 (MLH1), MutS homolog 2 and 3 (MSH2 and MSH3) and exonuclease 1 (EXO1) proteins are crucial to identify CDDP-induced DNA lesion and enable its removal [4–7].

Variations in toxicity and response to therapy and/or in survival were seen in patients with lung [8–11], pancreatic [12], breast [13, 14], laryngeal [15], cervical [16], and ovarian [17] cancer treated with CDDP-based schemes and/or RT, which were attributed to abnormalities in production or function of proteins encoded by single nucleotide polymorphisms (SNPs) in genes of MMR pathway. In fact, variant “A”, “G”, “A”, and “A” alleles of *MLH1* c.-93G>A (rs1800734) [18], *MSH2* c.211+9C>G (rs2303426) [19], *MSH3* c.3133G>A (rs26279) [20], and *EXO1* c.1765G>A (rs1047840) [21], reduce levels of expressed protein compared with respective wild-type alleles, and have reduced DNA repair as consequence. It was also observed association of wild-type “C” allele of *EXO1* c.2270C>T (rs9350) with decreased DNA repair, possibly due to its influence in preventing EXO1 involvement in the complex with MMR proteins [21].

Recently, we retrospectively analyzed *MLH1* rs1800734, *MSH2* rs2303426, *MSH3* rs26279, *EXO1* rs1047840, and *EXO1* rs9350 SNPs in HNSCC subjects that received concurrent chemoradiotherapy with CDDP/carboplatin as neoadjuvant, adjuvant or definitive treatment, and observed that patients with wild-type “GG” genotypes of *MSH3* rs26279 and *EXO1* rs1047840 presented shorter relapse-free survival (RFS) and overall survival (OS) compared to others; however, side effects and response to therapy were not evaluated in study [20]. We investigated in this new prospective study the roles of the above mentioned SNPs in modulation of toxicity and response to therapy of HNSCC patients treated exclusively with CDDP chemoradiation.

## RESULTS

### HNSCC patients

The clinicopathological aspects of 90 patients enrolled in study are presented in Table 1.

**Table 1:** Clinical and tumor aspects of 90 patients with head and neck squamous cell carcinoma

Variable	Median (range) or *N* (%)
**Age (years)**	56 (27–74)
**Gender**	
Male	83 (92.2)
Female	7 (7.8)
**Body mass index (kg/m**^**2**^**)**	19 (13–31)
**Tobacco consumption**	
Smokers	88 (97.8)
Nonsmokers	2 (2.2)
**Alcohol consumption**	
Drinkers	83 (92.2)
Abstainers	7 (7.8)
**Tumor location**	
Oral cavity	12 (13.3)
Pharynx	55 (61.1)
Larynx	23 (25.6)
Histological grade^*^	
Well + moderately	60 (82.2)
Poorly + undifferentiated	13 (17.8)
**Tumor stage**	
I + II	6 (6.7)
III + IV	84 (93.3)
Human papillomavirus type 16^*^	
Positive	0 (0.0)
Negative	57 (100.0)

N: number of patients. ^*^The number of patients differed from the total quoted in the study (*N* = 90) because it was not possible to obtain consistent information about histological grade and human papillomavirus type 16 status in some cases.

Sixty-eight patients were treated with three CDDP administrations, and 22 patients were treated with only two CDDP injections, because they presented hematologic or renal consistent toxicities. Median cumulative amount of administrated CDDP was 265 mg (range: 100–616). Adherence to antiemetics was medium or high in most of the patients (97.7%).

Grade 2 or grade 3 nausea and grade 2 to grade 4 vomiting were seen in about two-thirds and one-third of cases, respectively. It was observed that one-third to half of cases presented grade 2 to grade 4 cytopenias, and half of cases had grade 2 to grade 5 nephrotoxicity or grade 2 to grade 4 ototoxicity. All available patients obtained complete response (CR) (*N* = 15), partial response (PR) (*N* = 53) or stable disease (SD) (*N* = 5) with treatment (Supplementary Table 1). The mean value (±SD) of CDDP found in urine was 237.0 ug/mL ± 116.2.

Cases were followed up for a median period of 21 months (range: 3.0–74). The assessed probabilities of 24-months event-free survival (EFS) and OS were 35.0% and 40.0%, respectively. In October 2017, 23 patients survived, 6 of them with disease and 17 without disease; and 67 patients died, 59 of them by tumor impacts and 8 by other causes.

Hardy-Weinberg equilibrium (HWE) was confirmed at *MLH1* rs1800734 (χ^2^ = 2.56, *P* = 0.11), *MSH2* rs2303426 (χ^2^ = 0.73, *P* = 0.39), *MSH3* rs26279 (χ^2^ = 1.54, *P* = 0.21), *EXO1* rs1047840 (χ^2^ = 0.42, *P* = 0.52), and rs9350 (χ^2^ = 2.80, *P* = 0.09) loci in patients’ samples. A linkage disequilibrium (LD) between *EXO1* rs1047840 and *EXO1* rs9350 (D’ = 19%) was found in our sample, and two *EXO1* haplotypes (GT, AC) were included in further analyses due to possible clinical significance and frequency greater than 10%.

### Genetic polymorphisms, side effects, response rate and prognosis

The genotypes of all SNPs and haplotypes of *EXO1* rs1047840 and rs9350 in 90 studied patients stratified by toxicities and responses to therapy are presented in Table 2.

**Table 2:** *MLH1* rs1800734*, MSH2* rs2303426*, MSH3* rs26279*, EXO1* rs1047840 and *EXO1* rs9350 single nucleotide polymorphism genotypes and *EXO1* haplotypes in 90 patients with head and neck squamous cell carcinoma stratified by toxicity and response rate to concurrent chemoradiotherapy

Variable	Nephrotoxicity		Ototoxicity		Response rate			
	G0 + G1 *N* (%)	G2–G5 *N* (%)	G0 + G1 *N* (%)	G2–G4 *N* (%)	CR + PR *N* (%)	SD *N* (%)	CR *N* (%)	PR + SD *N* (%)
***MLH1* rs1800734**								
GG + GA	35 (52.2)	32 (47.8)	36 (52.2)	33 (47.8)	66 (93.0)	5 (7.0)	15 (21.1)	56 (78.9)
AA	1 (50.0)	1 (50.0)	0 (0.0)	1 (100.0)	2 (100.0)	0 (0.0)	0 (0.0)	2 (100.0)
*P*-value	0.99		0.99		0.99		0.99	
OR (95% CI)	NE		NE		NE		NE	
GG	20 (48.8)	21 (51.2)	20 (47.6)	22 (52.4)	42 (95.5)	2 (4.5)	9 (20.5)	35 (79.5)
GA + AA	16 (57.1)	12 (42.9)	16 (57.1)	12 (42.9)	26 (89.7)	3 (10.3)	6 (20.7)	23 (79.3)
*P*-value	0.29		0.46		0.10		0.47	
OR (95% CI)	1.76 (0.63–4.93)		1.48 (0.53–4.13)		0.13 (0.01–1.50)		0.58 (0.13–2.55)	
***MSH2* rs2303426**								
CC + CG	29 (51.8)	27 (48.2)	27 (48.2)	29 (51.8)	54 (91.5)	5 (8.5)	10 (16.9)	49 (83.1)
GG	7 (53.8)	6 (46.2)	9 (64.3)	5 (35.7)	14 (100.0)	0 (0.0)	5 (35.7)	9 (64.3)
*P*-value	0.80		0.59		0.99		0.39	
OR (95% CI)	1.18 (0.33–4.24)		1.43 (0.39–5.24)		NE		2.01 (0.41–9.96)	
CC	11 (61.1)	7 (38.9)	10 (55.6)	8 (44.4)	16 (94.1)	1 (5.9)	4 (23.5)	13 (76.5)
CG + GG	25 (49.0)	26 (51.0)	26 (50.0)	26 (50.0)	52 (92.9)	4 (7.1)	11 (19.6)	45 (80.4)
*P*-value	0.44		0.38		0.80		0.26	
OR (95% CI)	0.64 (0.21–1.98)		0.59 (0.18–1.93)		1.37 (0.12–15.71)		0.42 (0.10–1.90)	
***MSH3* rs26279**								
GG + GA	31 (49.2)	32 (50.8)	31 (49.2)	32 (50.8)	65 (95.6)	3 (4.4)	14 (20.6)	54 (79.4)
AA	5 (83.3)	1 (16.7)	5 (71.4)	2 (28.6)	3 (60.0)	2 (40.0)	1 (20.0)	4 (80.0)
*P*-value	0.26		0.04^3^		0.08		0.75	
OR (95% CI)	3.75 (0.38–37.03)		10.29 (1.06–100.21)		0.11 (0.01–1.31)		1.49 (0.12–18.17)	
GG	13 (35.1)	24 (64.9)	18 (46.2)	21 (53.8)	39 (95.1)	2 (4.9)	8 (19.5)	33 (80.5)
GA + AA	23 (71.9)	9 (28.1)	18 (58.1)	13 (41.9)	29 (90.6)	3 (9.4)	7 (21.9)	25 (78.1)
*P*-value	0.007^1^		0.13		0.61		0.56	
OR (95% CI)	4.27 (1.48–12.34)		2.28 (0.79–6.58)		0.60 (0.08–4.42)		1.48 (0.39–5.59)	
***EXO1* rs1047840**								
GG + GA	34 (55.7)	27 (44.3)	33 (52.4)	30 (47.6)	60 (92.3)	5 (7.7)	14 (21.5)	51 (78.5)
AA	2 (25.0)	6 (75.0)	3 (42.9)	4 (57.1)	8 (100.0)	0 (0.0)	1 (12.5)	7 (87.5)
*P*-value	0.07		0.61		0.99		0.99	
OR (95% CI)	4.88 (0.86–27.55)		0.64 (0.12–3.47)		NE		NE	
GG	18 (56.3)	14 (43.8)	19 (61.3)	12 (38.7)	31 (93.9)	2 (6.1)	11 (33.3)	22 (66.7)
GA + AA	18 (48.6)	19 (51.4)	17 (43.6)	22 (56.4)	37 (92.5)	3 (7.5)	4 (10.0)	36 (90.0)
*P*-value	0.35		0.30		0.83		0.01^5^	
OR (95% CI)	1.63 (0.59–4.51)		0.58 (0.21–1.62)		0.79 (0.10–6.56)		9.55 (1.56–58.60)	
***EXO1* rs9350**								
CC + CT	36 (52.2)	33 (47.8)	36 (51.4)	34 (48.6)	68 (93.2)	5 (6.8)	15 (20.5)	58 (79.5)
TT	0 (0.0)	0 (0.0)	0 (0.0)	0 (0.0)	0 (0.0)	0 (0.0)	0 (0.0)	0 (0.0)
*P*-value	0.99		0.99		0.99		0.99	
OR (95% CI)	NE		NE		NE		NE	
CC	21 (42.0)	29 (58.0)	30 (57.7)	22 (42.3)	49 (94.2)	3 (5.8)	12 (23.1)	40 (76.9)
CT	15 (78.9)	4 (21.1)	6 (33.3)	12 (66.7)	19 (90.5)	2 (9.5)	3 (14.3)	18 (85.7)
*P*-value	0.02^2^		0.06		0.60		0.27	
OR (95% CI)	4.69 (1.34–16.44)		4.03 (1.17–13.93)		0.59 (0.08–4.30)		0.38 (0.07–2.15)	
***EXO1 + EXO1***								
GT	14 (87.5)	2 (12.5)	5 (31.3)	11 (68.7)	16 (88.9)	2 (11.1)	3 (16.7)	15 (83.3)
Other haplotypes	22 (41.5)	31 (58.5)	31 (57.4)	23 (42.6)	52 (94.5)	3 (5.5)	12 (21.8)	43 (78.2)
*P*-value	0.06		0.03^4^		0.49		0.71	
OR (95% CI)	3.11 (1.85–44.75)		4.00 (1.11–14.48)		0.49 (0.07–3.72)		0.71 (0.13–4.07)	
AC	18 (48.6)	19 (51.4)	17 (43.6)	22 (56.4)	37 (92.5)	3 (7.5)	4 (10.0)	36 (90.0)
Other haplotypes	18 (56.3)	14 (43.7)	19 (61.3)	12 (38.7)	31 (93.9)	2 (6.1)	11 (33.3)	22 (66.7)
*P*-value	0.35		0.30		0.74		0.02^6^	
OR (95% CI)	0.61 (0.22–1.70)		0.58 (0.21–1.62)		0.70 (0.08–5.90)		9.55 (1.56–58.60)	

G: grade of toxicity; N: number of patients; CR: complete response; PR: partial response; SD: stable disease; OR: odds ratio; CI: confidence interval; NE: not evaluated. ORs were adjusted by age, cumulative dose of cisplatin and body mass index in analyses of nephrotoxicity, by age, cumulative dose of cisplatin and tumor location in analyses of ototoxicity for age, cumulative dose of cisplatin, body mass index, tumor location and grade in analyses of response rate. The total number of patients differed from the total quoted in the study (*N* = 90) because it was not possible to obtain consistent information about response rate and toxicities in some cases; ^1^*P* bootstrap = 0.006, ^2^*P* bootstrap = 0.005, ^3^*P* bootstrap = 0.01, ^4^*P* bootstrap = 0.02, ^5^*P* bootstrap = 0.007, ^6^*P* bootstrap = 0.005.

Patients with *MSH3* GG genotype had more frequently grade 2 to grade 5 nephrotoxicity than those with GA or AA genotype (64.9% *vs*. 28.1%); it was observed a 4.27-fold increased risk of substantial nephrotoxicity with treatment in patients with *MSH3* GG genotype. The frequency of *MSH3* GG or GA genotype was higher than AA in subjects with grade 2 to grade 4 ototoxicity (50.8% *vs*. 28.6%); after treatment, it was observed a 10.29-fold increased risk of substantial ototoxicity in patients with GG or GA genotype when compared to those with AA genotype.

**Figure 1:**
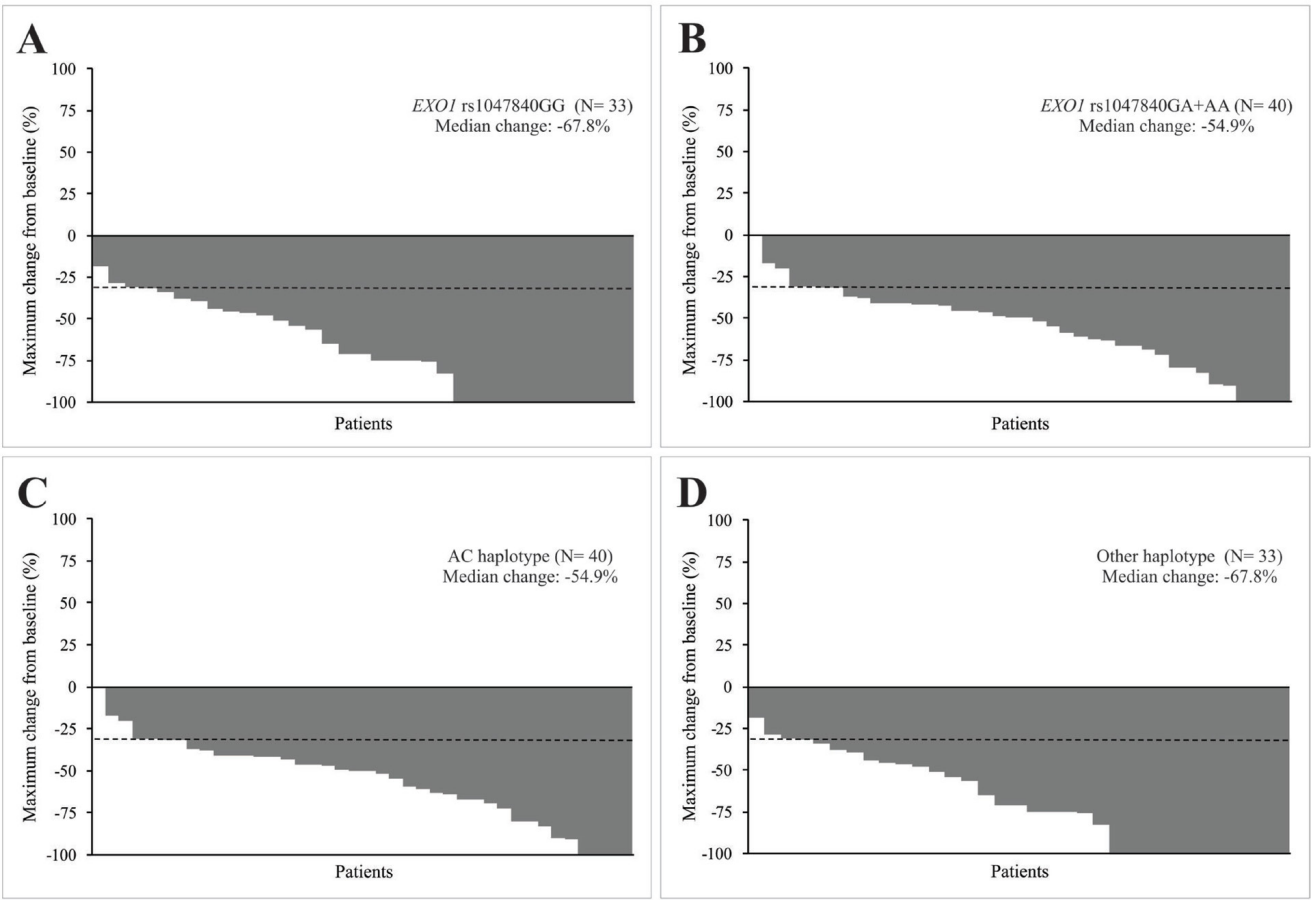
Characteristics of response to concurrent cisplatin chemoradiotherapy of head and neck squamous cell carcinoma (HNSCC) patients Waterfall plots indicate the maximum change from baseline in the sum of reference diameters of target lesion in patients with *EXO1* rs1047840 GG and GA or AA genotypes (**A** and **B**) an in patients with AC haplotype (variant allele of *EXO1* rs1047840 and wild-type allele of *EXO1* rs9350) and other haplotypes (**C** and **D**). The dashed lines indicate a 30% reduction in the tumor burden in the target lesion, as defined by Response Evaluation Criteria in Solid Tumors version 1.1.

*EXO1* rs1047840 GA or AA genotype was more common than GG genotype (90.0% *vs.* 66.7%) in patients with PR or SD; 9.55 more chances of presenting PR or SD instead of CR to chemoradiation were observed in patients with GA or AA genotype when compared to the ones with GG genotype. The median reduction in diameters of target lesions after chemoradiation was also less pronounced in patients with GA or AA genotype than in those with GG genotype (−54.9% *vs.* −67.8%) (Figure 1A, Figure 1B). *EXO1* rs9350 CC genotype was more common than CT or TT genotype (58.0% *vs.* 21.1%) in patients with grade 2 to grade 5 nephrotoxicity; it was observed a 4.69-fold increased risk of substantial nephrotoxicity in subjects with CC genotype after treatment, when compared to those with the remaining genotypes. An excess of GT haplotype (wild-type and variant alleles of *EXO1* rs1047840 and rs9350, respectively) was seen in subjects presenting grade 2 to grade 4 ototoxicity (68.7% *vs.* 42.6%) compared to those with other haplotypes; a 4.00-fold increased risk of substantial ototoxicity was observed in carriers of GT haplotype after chemoradiation, when compared to patients with other haplotypes. The AC haplotype (variant allele and wild-type allele of *EXO1* rs1047840 and rs9350, respectively) was more frequent than other haplotypes in subjects who obtained PR or SD (90.0 *vs.* 66.7%); it was observed a 9.55-fold increased risk of achieving PR or SD instead of CR in carriers of AC haplotype after treatment, when compared to patients with other haplotypes. The median reduction in diameters of target lesions after chemoradiation was also less pronounced in patients with AC haplotype compared to others (−54.9% *vs.* −67.8%) (Figure 1C, Figure 1D).

No associations between SNPs and haplotypes were observed in subjects classified by nausea, vomiting, cytopenias and urinary CDDP (Supplementary Table 2).

At 24 months of follow-up, shorter EFS (32.7% *vs.* 83.3%, *P* = 0.01) and OS (35.7% *vs.* 100.0%, *P* = 0.009) were seen in patients with advanced tumors compared to those with localized tumors (Kaplan-Meier estimates). A shorter EFS and OS were observed in those patients in uni and multivariate Cox analyses. No associations of *MLH1* rs1800734, *MSH2* rs2303426, *MSH3* rs26279, *EXO1* rs1047840, *EXO1* rs9350 SNPs, and *EXO1* haplotypes were seen in survival analyses of HNSCC patients (Supplementary Table 3).

## DISCUSSION

Clinical characteristics of patients, aspects of tumor, toxicity and response to chemoradiation, and survival rates found in the present study were similar to those previously described [1, 22, 23], indicating that our sample was satisfactory for investigations of new prognostic factors in HNSCC. None of our available patients had undergone HPV infection, as previously described in Brazilian HNSCC patients [24, 25]; this finding indicates that tobacco and alcohol were the most important factors related to tumor onset in our cases.

We found that *MSH3* rs26279 GG and GG or GA genotypes were associated with pronounced nephrotoxicity and ototoxicity, respectively. *MSH3* rs26279 altered radiosensitivity in breast cancer patients [13], response to platinum-based therapy and survival in lung cancer patients [9], response to CDDP chemoradiation and hematological toxicity in a unique case of laryngeal cancer [15] and survival in HNSCC patients treated by CDDP and RT [20], but its roles in nephrotoxicity and ototoxicity were not described in studies. We identified higher level of mRNA in *MSH3* rs26279 GG individuals compared to those with GA or AA genotype in a previous study [20]. It was proposed that increased expression of MSH3 sequesters nuclear MSH2 and favors formation of MutSβ heterodimer, having a drastic change in MutSα and MutSβ proportion and reduction of efficiency in repairing base-base mismatches as consequence [26]. Thus, renal tubular cells and outer hair cells in carriers of “G” allele may be able to undergo to apoptosis in response to DNA damages due to CDDP and RT, with consequent nephrotoxicity and ototoxicity.

*EXO1* rs1047840 GA or AA genotype and AC haplotype of *EXO1* rs1047840 and rs9350 SNPs were associated with worst response to chemoradiation in this study. *EXO1* rs1047840 GA genotype appeared to contribute to a CR and marked hematological toxicity seen in a patient with laryngeal cancer treated with CDDP and RT [15], and variant allele “A” of *EXO1* rs1047840 was also associated with a better response rate in patients with cervical carcinoma [16]. Jin *et al.* (2008) [21] proposed that *EXO1* rs1047840 variant “A” allele reduces the amount of mRNA of *EXO1*, and consequently decreases EXO1 protein levels and MMR activity, since it is fundamental for excision of DNA injuries initiated by CDDP, activating apoptosis [27, 28]. *EXO1* rs9350 determines change of proline to leucine in codon 757 of EXO1 protein [29]. Proline tends to destabilize α-helices due to the lack of a backbone hydrogen bond and steric constraints [30], implying that the presence of this aminoacid could influence protein-protein interaction, especially with MSH2, resulting in incomplete repair of DNA lesions and apoptosis of damaged cells [29]. Thus, we expected to obtain a better response to therapy in carriers of variant “A” allele of *EXO1* rs1047840 and wild-type “C” allele of rs9350 SNP, but the opposite result was found in this study. However, decrease of EXO1 expression in human fibroblasts or mouse embryonic fibroblasts caused a delay in DNA damage-induced apoptosis, and EXO1 may have a main role in caspase-3 cleavage, DNA fragmentation and cytochrome c release, participating in crucial phases of apoptosis; thus, SNPs in *EXO1* may not only imply in DNA repair, but may also favor cell survival by apoptotic defects [31]. Since the roles of the mentioned SNPs in the treatment of HNSCC patients are still unknown, our data suggest that they are associated with a worst response to CDDP chemoradiation.

We observed that *EXO1* rs9350 CC genotype was associated with pronounced nephrotoxicity and GT haplotype of *EXO1* rs1047840 and rs9350 SNPs was associated with pronounced ototoxicity. Decrease in DNA repair was previously attributed to wild-type “C” allele of *EXO1* rs9350, possibly due to its influence in protein-protein interaction, preventing EXO1 involvement in the complex with MMR proteins [21]. Thus, renal tubular cells in patients with CC genotype may undergo to apoptosis in response to DNA damages induced by CDDP and RT, with consequent nephrotoxicity. However, the association of wild-type “G” and variant “T” alleles of *EXO1* rs1047840 and rs9350 SNPs, associated with increased DNA repair [21], with ototoxicity was not expected in this study; these apparent controversial findings could be attributed to different roles of *EXO1* alleles, especially rs9350 “C”, which may be specific in distinct tissues/organs [32].

No association of analyzed genotypes and haplotypes with survival of 90 HNSCC was seen in this study. *MSH3* rs26279 and *EXO1* rs9350 altered survival of 180 lung cancer [9] and 602 lung cancer [10] patients treated with platinum-based chemotherapy. RFS and OS of 397 patients with HNSCC were also altered by *MSH3* rs26279 and *EXO1* rs1047840 SNPs in a previous study conducted by our group [20]. Differences of results could be related to different tumor types, sample sizes and median times of follow-up, which was about 2.0 times higher in our previous study than in the present one.

In conclusion, for the first time, this present study shows preliminary indication that inherited variations promoted by *MSH3* rs26279, *EXO1* rs1047840 and *EXO1* rs9350, involved in DNA MMR pathway, may alter side effects and response CDDP and RT in HNSCC patients. We are aware that further analysis in a larger number of patients and functional analyses of relevant SNPs will be required to confirm results and elucidate their roles in disease. We believe that these results may contribute to the future personalized treatment of HNSCC patients, possibly with the use of varying CDDP doses and protective agents against CDDP-induced nephro- and ototoxicity [33, 34].

## MATERIALS AND METHODS

Ninety HNSCC patients at diagnosis, seen at the University of Campinas from June 2011 to February 2014, were enrolled in this prospective study. Patients were chosen to CDDP chemoradiation as definitive treatment according to the following inclusion criteria: did not accepted surgical resolution facing expected anatomic or functional sequels, locoregional unresectable tumor, or a strategy of organ preservation. Declaration of Helsinki was conducted and the institutional Ethics Committee approved this study (n. 274/2011), and all patients provided written informed consent.

Patients were separated as smokers *vs*. nonsmokers and drinkers *vs*. abstainers [35]. HNSCC was diagnosed and staged based on conventional criteria [36, 37]. P16 immunohistochemistry and *in situ* hybridization were performed in tumor fragments, aiming to test the presence of human papillomavirus type 16 (HPV 16) [38, 39].

Patients were treated with a starting dose of 80–100 mg/m^2^ of “in bolus” intravenous injection of CDDP on 1st, 22th and 43th days concomitant with RT (70 Gy; 35 sessions); lower dose of CDDP (50–75 mg/m^2^) was delivered in second and/or third infusion in patients who presented toxicity with the first administration of the agent [1, 40]. As hydration and antiemetic protocols, they received intravenous saline solution, mannitol, ondansetron and dexamethasone before CDDP administration, as well as intravenous physiological saline and oral dexamethasone and metoclopramide during three days after each CDDP infusion [41, 42]. Antiemetic adherence was analyzed [43].

Nausea and vomiting were assessed immediately after each CDDP infusion and in the four following days. Cytopenias were evaluated with complete blood counts performed after each CDDP administration. Nephrotoxicity was analyzed using glomerular filtration rate measured by ^51^Cr-EDTA and ototoxicity was assessed by audiometric exams, both performed pre and post chemoradiation, respectively [44]. The worst grades of toxicities seen during the entire treatment were considered for analyses.

Response to therapy was categorized as CR, PR, SD or progressive disease (PD) [45]. Immediately after each CDDP administration, 48-hours urine collection was performed for estimation of CDDP by high-performance liquid chromatography assay [46]; the sum of cumulative measurement of urinary CDDP estimates acquired after each CDDP infusion was considered the final concentration.

Subjects with PR after treatment or tumor relapse and good clinical condition were selected for surgical tumor resection; palliative intravenous methotrexate was indicated to patients with unfavorable clinical performance [47].

Genotyping was performed by polymerase chain reaction (PCR) and enzymatic digestion or PCR real-time assay, using DNA from patients’ peripheral blood samples as reported for *MLH1* rs1800734 [48], *MSH2* rs2303426 [49], *MSH3* rs26279 [20], *EXO1* rs1047840 and rs9350 [27]. Total of 15% of genotypes were carried out by two independent experiments with total concordance.

For goodness-of-fit test was used chi-square (*χ*^2^) statistics to evaluate HWE. The Haploview 4.2 software was used to perform pairwise LD analyses of *EXO1* haplotypes. To analyze differences between groups, *χ*^*2*^ or Fisher’s exact test were used. To obtain adjusted odds ratio (OR) values and to assess associations among SNPs, toxicity and response to treatment, and urinary CDDP, model of logistic regression was used. To ensure the stability of model was used the bootstrapping (*N* = 1,000) based on repeatedly random sampling, applying the bias-corrected and accelerated method [50].

EFS and OS were computed from the date of diagnosis to the first relapse, death from disease or last follow-up, and from the date of diagnosis until the death, resulting from any cause, or last follow-up, respectively. Kaplan-Meier method was used to analyze EFS and OS. Multivariate Cox regression was used to estimate hazard ratios (HRs) adjusted for possible discrepancies in clinical aspects (*P*-values ≤ 0.10 in univariate Cox regression).

All statistical tests were done using the SPSS 21.0 software (SPSS Incorporation, IL, USA), and two-sided significance was achieved when *P-*values were < 0.05.

## SUPPLEMENTARY MATERIALS TABLES





## Abbreviations

^51^Cr-EDTA: chromium-51 labeled ethylenediamine tetraacetic acid
95% CI: 95% confidence interval
CDDP: cisplatin
CR: complete response
CreCl: creatinine clearance
EFS: event-free survival
D’: disequilibrium coefficient
DNA: deoxyribonucleic acid
EXO1: exonuclease 1
FAPESP: São Paulo Research Foundation
G: grade of toxicity
Gy: gray
HNSCC: head and neck squamous cell carcinoma
HPV 16: human papillomavirus type 16
HR: hazard ratio
HWE: Hardy-Weinberg equilibrium
LD: linkage disequilibrium
MDR: Dimensionality Reduction Model
MLH1: MutL homolog 1
MMR: mismatch repair
MSH2: MutS homolog 2
MSH3: MutS homolog 3
N: number of patients
NCI: National Cancer Institute
NE: not evaluated
OR: odds ratio
OS: overall survival
PCR: polymerase chain reaction
PD: progressive disease
PR: partial response
RECIST 1.1: Response Evaluation Criteria in Solid Tumors 1.1
RT: radiotherapy
SD: stable disease
SNP: single nucleotide polymorphism
χ^2^: chi-square
