# Supplementary material for: Polymorphisms in DNA mismatch repair pathway genes predict toxicity and response to cisplatin chemoradiation in head and neck squamous cell carcinoma patients
Source: Oncotarget. 2018 Jul 3;9(51):29538–47. doi: 10.18632/oncotarget.25268 (PMC6049861; doi:10.18632/oncotarget.25268)
Supplement: Supplementary file 1 [file oncotarget-09-29538-s001.pdf]

Polymorphisms in DNA mismatch repair pathway genes predict toxicity and response to cisplatin chemoradiation in head and neck squamous cell carcinoma patients

SUPPLEMENTARY MATERIALS

Supplementary Table 1: Toxicities and responses to chemoradiotherapy in 90 patients with head and neck squamous cell carcinoma

Variable	Mild, ideal		Moderate or severe, non-ideal	
	Grade of toxicity or type of response	*N* (%)	Grade of toxicity or type of response	*N* (%)
**Gastrointestinal toxicity**				
Nausea	G0 + G1	37 (42.0)	G2 + G3	51 (58.0)
Vomiting	G0 + G1	59 (67.0)	G2 + G3 + G4	29 (33.0)
**Hematologic toxicity**				
Anemia	G0 + G1	37 (44.0)	G2 + G3 + G4	47 (56.0)
Leukopenia	G0 + G1	47 (56.0)	G2 + G3 + G4	37 (44.0)
Neutropenia	G0 + G1 + G2	67 (79.8)	G3 + G4	17 (20.2)
Lymphopenia	G0 + G1 + G2	42 (50.0)	G3 + G4	42 (50.0)
Thrombocytopenia	G0	54 (64.3)	G1 + G2 + G3 + G4	30 (35.7)
**Nephrotoxicity**	G0 + G1	36 (52.2)	G2 + G3 + G4 + G5	33 (47.8)
**Ototoxicity**	G0 + G1	36 (51.4)	G2 + G3 + G4	34 (48.6)
**Response rate**	CR + PR	68 (93.2)	SD	5 (6.8)
	CR	15 (20.5)	PR + SD	58 (79.5)

N: number of patients; G: grade of toxicity; CR: complete response; PR: partial response; SD: stable disease. Toxicities and responses to chemoradiation were classified using the Common Terminology Criteria for Adverse Events of the National Cancer Institute and RECIST guideline, respectively. The total number of patients analyzed in distinct variables differed from the total quoted in study ($N = 90$) because it was not possible to obtain consistent information about nausea, vomiting, hematologic exams, glomerular filtration rate, audiometry test or response rate after chemoradiotherapy in some cases.

Supplementary Table 2: *MLH1*, *MSH2*, *MSH3* and *EXO1* single nucleotide polymorphism genotypes and haplotypes in 90 head and neck squamous cell carcinoma patients stratified by nauseas, vomiting, hematologic toxicities to chemoradiotherapy and urinary cisplatin. See Supplementary_Table_2

Supplementary Table 3: Association of clinical and tumor characteristics, *MLH1*, *MSH2*, *MSH3*, and *EXO1* single nucleotide polymorphism genotypes and *EXO1* haplotypes with survival of 90 head and neck squamous cell carcinoma patients treated with chemoradiotherapy

Variable	Univariate analysis					
	Event-free survival			Overall survival		
	N with event/ *N* total	*P* value	HR (95% CI)	*N* with event/*N* total	*P* value	HR (95% CI)
**Age (years)**						
≤ 56	33/46	0.33	1.27 (0.77–2.10)	32/46	0.86	1.04 (0.63–1.71)
> 56	29/44			31/44		
**Gender**						
Male	56/83	0.21	1.70 (0.73–3.96)	59/83	0.68	1.23 (0.44–3.42)
Female	6/7			4/7		
**Tobacco consumption**						
Smokers	60/88	0.12	3.05 (0.73–12.67)	62/88	0.73	1.40 (0.19–10.19)
Nonsmokers	2/2			1/2		
**Alcohol consumption**						
Drinkers	59/83	0.31	1.82 (0.57–5.81)	60/83	0.16	2.30 (0.71–7.39)
Abstainers	3/7			3/7		
**Tumor location**						
Oral cavity/oropharynx	38/51	0.37	1.26 (0.75–2.10)	38/51	0.36	1.26 (0.76–2.09)
Hypopharynx/larynx	24/39			25/39		
**Histological grade**						
Well/moderately	41/60	0.36	1.39 (0.67–2.89)	42/60	0.60	1.20 (0.58–2.48)
Poorly/undifferentiated	9/13			9/13		
**Tumor stage**						
I + II	1/6	0.05	7.09 (0.98–51.26)*	1/6	0.03	8.08 (1.11–58.49)*****
III + IV	61/84			62/84		
***MLH1* rs1800734**						
GG+GA	60/88	0.30	2.09 (0.50–8.66)	62/88	0.81	1.26 (0.17–9.16)
AA	2/2			1/2		
GG	27/51	0.62	1.13 (0.68–1.87)	36/51	0.98	1.00 (0.60–1.65)
GA+AA	35/39			27/39		
***MSH2* rs2303426**						
CC+CG	46/71	0.81	1.07 (0.60–1.89)	49/71	0.75	1.09 (0.61–1.95)
GG	16/19			14/19		
CC	14/22	0.36	1.31 (0.72–2.38)	15/22	0.64	1.15 (0.63–2.08)
CG+GG	48/68			48/68		
***MSH3* rs26279**						
GG+GA	55/81	0.51	1.30 (0.59–2.86)	58/81	0.35	1.54 (0.61–3.84)
AA	7/9			5/9		
GG	35/50	0.87	1.04 (0.63–1.72)	35/50	0.95	1.01 (0.61–1.67)
GA+AA	27/40			28/40		
***EXO1* rs1047840**						
GG+GA	57/82	0.43	1.44 (0.57–3.60)	57/82	0.82	1.10 (0.47–2.56)
AA	5/8			6/8		
GG	31/40	0.09	1.54 (0.93–2.53)**	29/40	0.54	1.16 (0.71–1.91)
GA+AA	31/50			34/50		

***EXO1* rs9350**						
CC	43/63	0.63	1.14 (0.66–1.95)	42/63	0.27	1.33 (0.79–2.25)
CT	19/27			21/27		
***EXO1* + EXO1**						
GT	18/24	0.26	1.36 (0.78–2.36)	19/24	0.15	1.47 (0.86–2.52)
Other haplotypes	44/66			44/66		
AC	31/50	0.09	1.54 (0.93–2.53)****	34/50	0.54	1.16 (0.71–1.91)
Other haplotypes	31/40			29/40		

N: number of patients; HR: hazard ratio; CI: confidence interval; NE: not evaluated. Tumor stage was defined using the criteria of the American Joint Committee on Cancer. Results obtained in multivariate Cox analysis adjusted by tumor stage: **P* = 0.045, HR: 7.03, 95% CI: 0.97–50.82; ***P* = 0.09, HR: 1.52, 95% CI: 0.92–2.52; ****P* = 0.19, HR: 1.67, 95% CI: 0.77–3.65; *****P* = 0.09, HR: 1.52, 95% CI: 0.92–2.52; ******P* = 0.03, HR: 8.08, 95% CI: 1.11–58.49.
