# Supplementary material for: Polymorphisms in DNA mismatch repair pathway genes predict toxicity and response to cisplatin chemoradiation in head and neck squamous cell carcinoma patients
Source: Oncotarget. 2018 Jul 3;9(51):29538–47. doi: 10.18632/oncotarget.25268 (PMC6049861; doi:10.18632/oncotarget.25268)
Supplement: Supplementary file 2 [file oncotarget-09-29538-s002.docx]

**Supplementary Table 2:** ***MLH1*, *MSH2*, *MSH3* and *EXO1* single nucleotide polymorphism genotypes and haplotypes in 90 head and neck squamous cell carcinoma patients stratified by nauseas, vomiting, hematologic toxicities to chemoradiotherapy and urinary cisplatin**

| **Variable** | **Nausea** | | **Vomiting** | | **Anemia** | | | **Leukopenia** | | | **Neutropenia** | | **Thrombocytopenia** | | | **Urinary cisplatin** | |
| --- | --- | --- | --- | --- | --- | --- | --- | --- | --- | --- | --- | --- | --- | --- | --- | --- | --- |
|  | G0+G1 N (%) | G2+G3 N (%) | G0+G1N (%) | G2-G4 N (%) | G0+G1N (%) | G2 to G4N (%) | | G0+G1N (%) | G2 to G4 N (%) | | G0 to G2 N (%) | G3+G4N (%) | G0 N (%) | G1 to G4 N (%) | | < 237 ug/mL (%) | ≥ 237 ug/mL (%) |
| ***MLH1* rs1800734** |  |  |  |  |  |  | |  |  | |  |  |  |  | |  |  |
| GG+GA | 37 (43.0) | 49 (57.0) | 57 (66.3) | 29 (33.7) | 36 (43.9) | 46 (56.1) | | 46 (56.1) | 36 (43.9) | | 65 (79.3) | 17 (20.7) | 52 (63.4) | 30 (36.6) | | 30 (52.6) | 27 (47.4) |
| AA | 0 (0.0) | 2 (100.0) | 2 (100.0) | 0 (0.0) | 1 (50.0) | 1 (50.0) | | 1 (50.0) | 1 (50.0) | | 2 (100.0) | 0 (0.0) | 2 (100.0) | 0 (0.0) | | 1 (100.0) | 0 (0.0) |
| *P*-value | 0.99 | | 0.99 | | 0.99 | | | 0.99 | | | 0.99 | | 0.99 | | | 0.99 | |
| OR (95% CI) | NE | | NE | | NE | | | NE | | | NE | | NE | | | NE | |
| GG | 20 (39.2) | 31 (60.8) | 33 (64.7) | 18 (35.3) | 23 (46.9) | 26 (53.1) | | 30 (61.2) | 19 (38.8) | | 39 (79.6) | 10 (20.4) | 34 (69.4) | 15 (30.6) | | 18 (51.4) | 17 (48.6) |
| GA+AA | 17 (45.9) | 20 (54.1) | 26 (70.3) | 11 (29.7) | 14 (40.0) | 21 (60.0) | | 17 (48.6) | 18 (51.4) | | 28 (80.0) | 7 (20.0) | 20 (57.1) | 15 (42.9) | | 13 (56.5) | 10 (43.5) |
| *P*-value | 0.69 | | 0.66 | | 0.40 | | | 0.19 | | | 0.88 | | 0.26 | | | 0.37 | |
| OR (95% CI) | 1.20 (0.49-2.94) | | 1.24 (0.48-3.22) | | 0.68 (0.28-1.68) | | | 0.54 (0.22-1.34) | | | 1.09 (0.36-3.28) | | 0.59 (0.23-1.47) | | | 1.70 (0.54-5.34) | |
| ***MSH2* rs2303426** |  |  |  |  |  |  | |  |  | |  |  |  |  | |  |  |
| CC+CG | 29 (42.0) | 40 (58.0) | 46 (66.7) | 23 (33.3) | 28 (41.8) | 39 (58.2) | | 37 (55.2) | 30 (44.8) | | 52 (77.6) | 15 (22.4) | 41 (61.2) | 26 (38.8) | | 24 (50.0) | 24 (50.0) |
| GG | 8 (42.1) | 11 (57.9) | 13 (68.4) | 6 (31.6) | 9 (52.9) | 8 (47.1) | | 10 (58.8) | 7 (41.2) | | 15 (88.2) | 2 (11.8) | 13 (76.5) | 4 (23.5) | | 7 (70.0) | 3 (30.0) |
| *P*-value | 0.91 | | 0.78 | | 0.37 | | | 0.84 | | | 0.31 | | 0.27 | | | 0.23 | |
| OR (95% CI) | 1.06 (0.37-3.09) | | 1.17 (0.38-3.63) | | 1.64 (0.55-4.87) | | | 1.12 (0.37-3.37) | | | 2.27 (0.46-11.24) | | 2.00 (0.58-6.85) | | | 2.52 (0.56-11.37) | |
| CC | 12 (54.5) | 10 (45.5) | 14 (63.6) | 8 (36.4) | 10 (47.6) | 11 (52.4) | | 15 (71.4) | 6 (28.6) | | 19 (90.5) | 2 (9.5) | 14 (66.7) | 7 (33.3) | | 7 (46.7) | 8 (53.3) |
| CG+GG | 25 (37.9) | 41 (62.1) | 45 (68.2) | 21 (31.8) | 27 (42.9) | 36 (57.1) | | 32 (50.8) | 31 (49.2) | | 48 (76.2) | 15 (23.8) | 40 (63.5) | 23 (36.5) | | 24 (55.8) | 19 (44.2) |
| *P*-value | 0.14 | | 0.72 | | 0.65 | | | 0.09 | | | 0.18 | | 0.80 | | | 0.37 | |
| OR (95% CI) | 0.14 (0.16-1.28) | | 1.22 (.42-3.49) | | 0.79 (0.29-2.19) | | | 0.39 (0.13-1.16) | | | 0.34 (0.07-1.65) | | 0.88 (0.31-2.50) | | | 1.86 (0.53-6.53) | |
| ***MSH3* rs26279** |  |  |  |  |  |  | |  |  | |  |  |  |  | |  |  |
| GG+GA | 32 (40.5) | 47 (59.5) | 54 (68.4) | 25 (31.6) | 34 (45.3) | 41 (54.7) | | 41 (54.7) | 34 (45.3) | | 59 (78.7) | 16 (21.3) | 50 (66.7) | 25 (33.3) | | 27 (50.0) | 27 (50.0) |
| AA | 5 (55.6) | 4 (44.4) | 5 (55.6) | 4 (44.4) | 3 (33.3) | 6 (66.7) | | 6 (66.7) | 3 (33.3) | | 8 (88.9) | 1 (11.1) | 4 (44.4) | 5 (55.6) | | 4 (100.0) | 0 (0.0) |
| *P*-value | 0.26 | | 0.48 | | 0.66 | | | 0.25 | | | 0.60 | | 0.22 | | | 0.99 | |
| OR (95% CI) | 2.50 (0.51-12.31) | | 0.56 (0.11-2.77) | | 0.71 (0.15-3.28) | | | 2.55 (0.52-12.49) | | | 1.81 (0.20-16.30) | | 0.39 (0.09-1.72) | | | NE | |
| GG | 20 (41.7) | 28 (58.3) | 35 (72.9) | 13 (27.1) | 20 (43.5) | 26 (56.5) | | 26 (56.5) | 20 (43.5) | | 33 (71.7) | 13 (28.3) | 30 (65.2) | 16 (34.8) | | 22 (59.5) | 15 (40.5) |
| GA+AA | 17 (42.5) | 23 (57.5) | 24 (60.0) | 16 (40.0) | 17 (44.7) | 21 (55.3) | | 21 (55.3) | 17 (44.7) | | 34 (89.5) | 4 (10.5 | 24 (63.2) | 14 (36.8) | | 9 (42.9) | 12 (57.1) |
| *P*-value | 0.76 | | 0.24 | | 0.71 | | | 0.88 | | | 0.07 | | 0.85 | | | 0.12 | |
| OR (95% CI) | 1.15 (0.47-2.85) | | 0.56 (0.21-1.46) | | 1.18 (0.48-2.90) | | | 1.07 (0.44-2.62) | | | 3.19 (0.93-10.92) | | 0.92 (0.37-2.29) | | | 2.56 (0.79-8.33) | |
| ***EXO1* rs1047840** |  |  |  |  |  |  | |  |  | |  |  |  |  | |  |  |
| GG+GA | 34 (42.5) | 46 (57.5) | 53 (66.3) | 27 (33.8) | 35 (45.5) | 42 (54.5) | | 44 (57.1) | 33 (42.9) | | 61 (79.2) | 16 (20.8) | 49 (63.6) | 28 (36.4) | | 29 (55.8) | 23 (44.2) |
| AA | 3 (37.5) | 5 (62.5) | 6 (75.0) | 2 (25.0) | 2 (28.6) | 5 (71.4) | | 3 (42.9) | 4 (57.1) | | 6 (85.7) | 1 (14.3) | 5 (71.4) | 2 (28.6) | | 2 (33.3) | 4 (66.7) |
| *P*-value | 0.92 | | 0.52 | | 0.42 | | | 0.43 | | | 0.66 | | 0.73 | | | 0.33 | |
| OR (95% CI) | 0.93 (0.20-4.38) | | 1.75 (0.31-9.88) | | 0.49 (0.09-2.73) | | | 0.52 (0.11-2.57) | | | 1.64 (0.18-14.83) | | 1.35 (0.24-7.49) | | | 0.40 (0.07-2.47) | |
| GG | 14 (35.9) | 25 (64.1) | 26 (66.7) | 13 (33.3) | 17 (43.6) | 22 (56.4) | | 24 (61.5) | 15 (38.5) | | 31 (79.5) | 8 (20.5) | 26 (66.7) | 13 (33.3) | | 14 (51.9) | 13 (48.1) |
| GA+AA | 23 (46.9) | 26 (53.1) | 33 (67.3) | 16 (32.7) | 20 (44.4) | 25 (55.6) | | 23 (51.1) | 22 (48.9) | | 36 (80.0) | 9 (20.0) | 28 (62.2) | 17 (37.8) | | 17 (54.8) | 14 (45.2) |
| *P*-value | 0.35 | | 0.97 | | 0.99 | | | 0.36 | | | 0.99 | | 0.74 | | | 0.99 | |
| OR (95% CI) | 1.53 (0.63-3.75) | | 0.98 (0.39-2.49) | | 0.99 (0.41-2.42) | | | 0.66 (0.27-1.61) | | | 1.01 (0.34-2.95) | | 0.86 (0.35-2.13) | | | 1.01 (0.35-2.94) | |
| ***EXO1* rs9350** |  |  |  |  |  |  | |  |  | |  |  |  |  | |  |  |
| CC | 26 (42.6) | 35 (57.4) | 39 (63.9) | 22 (36.1) | 27 (45.0) | 33 (55.0) | | 34 (56.7) | 26 (43.3) | | 47 (78.3) | 13 (21.7) | 37 (61.7) | 23 (38.3) | | 8 (44.4) | 10 (55.6) |
| CT+TT | 11 (40.7) | 16 (59.3) | 20 (74.1) | 7 (25.9) | 10 (41.7) | 14 (58.3) | | 13 (54.2) | 11 (45.8) | | 20 (83.3) | 4 (16.7) | 17 (70.8) | 7 (29.2) | | 23 (57.5) | 17 (42.5) |
| *P*-value | 0.93 | | 0.26 | | 0.79 | | | 0.73 | | | 0.55 | | 0.48 | | | 0.43 | |
| OR (95% CI) | 0.96 (0.37-2.50) | | 1.83 (0.64-5.26) | | 0.87 (0.33-2.32) | | | 0.84 (0.32-2.23) | | | 1.46 (0.42-5.09) | | 1.45 (0.52-4.08) | | | 1.59 (0.50-5.02) | |
| CC+CT | 37 (42.0) | 51 (58.0) | 59 (67.0) | 29 (33.0) | 37 (44.0) | | 47 (56.0) | 47 (56.0) | | 37 (44.0) | 67 (79.8) | 17 (20.2) | 54 (64.3) | | 30 (35.7) | 31 (53.4) | 27 (46.6) |
| TT | 0 (0.0) | 0 (0.0) | 0 (0.0) | 0 (0.0) | 0 (0.0) | | 0 (0.0) | 0 (0.0) | | 0 (0.0) | 0 (0.0) | 0 (0.0) | 0 (0.0) | | 0 (0.0) | 0 (0.0) | 0 (0.0) |
| *P-value* | 0.99 | | 0.99 | | 0.99 | | | 0.99 | | | 0.99 | | 0.99 | | | 0.99 | |
| OR (95% CI) | NE | | NE | | NE | | | NE | | | NE | | NE | | | NE | |
| ***EXO1 + EXO1*** |  |  |  |  |  |  | |  |  | |  |  |  |  | |  |  |
| GT | 10 (41.7) | 14 (58.3) | 10 (41.7) | 14 (58.3) | 9 (40.9) | 13 (59.1) | | 12 (54.5) | 10 (45.5) | | 19 (86.4) | 3 (13.6) | 17 (77.3) | 5 (22.7) | | 24 (55.8) | 19 (44.2) |
| Other haplotypes | 27 (42.2) | 37 (57.8) | 27 (42.2) | 37 (57.8) | 28 (45.2) | 34 (54.8) | | 35 (56.5) | 27 (43.5) | | 48 (77.4) | 14 (22.6) | 37 (59.7) | 25 (40.3) | | 7 (46.7) | 8 (53.3) |
| *P*-value | 0.99 | | 0.99 | | 0.73 | | | 0.79 | | | 0.34 | | 0.16 | | | 0.61 | |
| OR (95% CI) | 0.99 (0.37-2.68) | | 0.99 (0.37-2.68) | | 0.84 (0.31-2.29) | | | 0.87 (0.32-2.37) | | | 1.94 (0.50-7.61) | | 2.25 (0.73-6.94) | | | 0.73 (0.22-2.46) | |
| AC | 23 (46.9) | 26 (53.1) | 23 (46.9) | 26 (53.1) | 20 (44.4) | 25 (55.6) | | 23 (51.1) | 22 (48.9) | | 36 (80.0) | 9 (20.0) | 28 (62.2) | 17 (37.8) | | 14 (51.9) | 13 (48.1) |
| Other haplotypes | 14 (35.9) | 25 (64.1) | 14 (35.9) | 25 (64.1) | 17 (43.6) | 22 (56.4) | | 24 (61.5) | 15 (38.5) | | 31 (79.5) | 8 (20.5) | 26 (66.7) | 13 (33.3) | | 17 (54.8) | 14 (45.2) |
| *P*-value | 0.35 | | 0.35 | | 0.99 | | | 0.36 | | | 0.99 | | 0.74 | | | 0.99 | |
| OR (95% CI) | 1.53 (0.63-3.75) | | 1.53 (0.63-3.75) | | 0.99 (0.41-2.42) | | | 0.66 (0.27-1.61) | | | 1.01 (0.34-2.95) | | 0.86 (0.35-2.13) | | | 1.01 (0.54-2.94) | |

(G) grade of toxicity; (N) number of patients; (SD) standard deviation; (OR) odds ratio; (CI) confidence interval; (NE) not evaluated. Toxicities were graded by the Common Terminology Criteria for Adverse Events of the National Cancer Institute. The total number of patients differed from the total quoted in the study (N= 90) because it was not possible to obtain consistent information about hematologic exams and urinary cisplatin in some cases. ORs were adjusted by age, cumulative dose of cisplatin and antiemetic adherence to nausea and vomiting, age and cumulative dose of cisplatin to hematologic toxicities, and age and cumulative dose of cisplatin to urinary cisplatin
